# Predictors of prolonged length of hospital stay and in-hospital mortality in patients aged 1–24 months with acute bronchiolitis in Douala, Cameroon

**DOI:** 10.1186/s12887-024-04653-x

**Published:** 2024-02-29

**Authors:** Charlotte Eposse Ekoube, Emmanuel Heles Nsang, Patricia Épée, Edgar Mandeng Ma Linwa, Yolande Djike Puepi, Ritha Mbono Betoko, Diomède Noukeu Njinkui, Dominique Enyama, Dora Mbonjo Bitsie, Jeannette Disso Massako, Soumaiyatou Abba, Esther Eleonore Ngo Linwa, Calixte Ida Penda

**Affiliations:** 1 Faculty of Medicine and Pharmaceutical Sciences, University of Douala, Douala, Cameroon; 2 Paediatric unit, Douala Laquintinie Hospital, Douala, Cameroon; 3 Faculty of Health Sciences, University of Buea, Buea, Cameroon; 4 Faculty of Medicine and Pharmaceutical Sciences, University of Dschang, Dschang, Cameroon; 5 Faculty of Health Sciences, University of Bamenda, Bamenda, Cameroon

**Keywords:** Bronchiolitis, Predictors, Hospital stay, Mortality

## Abstract

**Introduction:**

In Cameroon, acute bronchiolitis has been reported as the third commonest lower respiratory infection and is usually associated with low mortality. Nonetheless, respiratory distress associated with non-adherence to management guidelines can prolong hospital stay. This study aimed to explore predictors of prolonged hospital stay (≥ 5 days) and mortality in patients aged < 2years hospitalised for acute bronchiolitis.

**Methodology:**

We conducted a retrospective cohort study at three paediatric units in the city of Douala, Cameroon. Factors associated with prolonged hospital stay and mortality were determined using multivariable linear regression model. Threshold for significance was set at *p* ≤ 0.05.

**Results:**

A total of 215 patients with bronchiolitis were included with mean age of 6.94 ± 5.71 months and M/F sex ratio of 1.39/1. Prolonged hospital stay was reported in 46.98% and mortality in 10.70% of patients hospitalised for bronchiolitis. Factors independently associated with prolonged hospital duration were oxygen administration [b = 0.36, OR = 2.35 (95% CI:1.16–4.74), *p* = 0.017], abnormal respiratory rate [b = 0.38, OR = 2.13 (1.00-4.55), *p* = 0.050] and patients presenting with cough [b = 0.33, OR = 2.35 (95% CI: 1.22–4.51), *p* = 0.011], and diarrhoea [b = 0.71, OR = 6.44 (95% CI: 1.6-25.86), *p* = 0.009] on admission. On the other hand, factors independently associated with mortality were age of the patient [b= -0.07, OR = 0.84 (95% CI: 0.74–0.97), *p* = 0.014] and oxygen administration [b = 1.08, OR = 9.64 (95% CI:1.16–79.85), *p* = 0.036]

**Conclusion:**

Acute bronchiolitis represented 1.24% of admissions and was common in the rainy season, in males and 3–11-month-old patients. Management guidelines were poorly respected. Prolonged length of stay was reported in half of the patients hospitalized and mortality was high, especially in younger patients and in patients receiving oxygen.

## Key message

Though the burden of acute bronchiolitis in Cameroon is still high, there is low adherence to management guidelines. Age and need for oxygen were associated with mortality.

## Introduction

Acute bronchiolitis is a seasonal viral respiratory tract infection, affecting children aged 1 to 24 months [1]. Multiple respiratory viruses such as rhinovirus, human metapneumovirus and parainfluenza viruses can cause acute bronchiolitis, however, the most common virus is the human Respiratory Syncytial Virus (RSV), which is responsible for more than 60% of cases [2–5]. Globally, RSV-caused infections affect roughly 33 million (21.6–50.3 million) of children under the age of five, and contribute to 3.2 million hospitalisations (range: 2.7–3.8 million), and 120,000 deaths (range: 94,000-149,000) each year [6]. The mean cost of hospitalisation is estimated at €3,973 per child < 1 year old [7]. It is clinically characterized by predominantly expiratory dyspnea, signs of respiratory distress, and wheezing which is sometimes audible at a distance [8]. The clinical course of this infection is variable, ranging from the mild forms managed on an ambulatory basis to more severe forms characterized by acute respiratory distress requiring hospitalization [8]. Mortality is less than 2% according to various reports [1, 9, 10].

In Cameroon, acute bronchiolitis has been reported as the third commonest lower respiratory infection [9]. Though consensual management guidelines for acute bronchiolitis are not yet known, the use of medications like bronchodilators and corticosteroids are not recommended [11–13]. In 2003, in France, adherence to these guidelines was reported at 6% of cases, which improved to 52.5% in 2013 [14, 15] with subsequent reduction in mortality [16]. Non-adherence to these guidelines have been associated with increased hospital length of stay [17] with its corollary of increased cost of care.

Only few reports in Sub-Saharan Africa have attempted to explore the burden and management of acute bronchiolitis. The aim of this work was to explore the hospital management practices with respect to recommended guidelines and analyse factors associated with prolonged length of stay and mortality in children hospitalized for acute bronchiolitis.

## Methodology

### Study design and study setting

This was a retrospective cohort study carried out in three hospitals in the city of Douala, the economic capital of Cameroon. Our study sites were the pediatric units of three hospitals: one is a second category hospital i.e. Laquintinie Hospital of Douala (HLD) and the two others are first category hospitals i.e., the General Hospital of Douala (HGD) and the Paediatric and Gynaecological Hospital of Douala (HGOPED).

### Study period

The study involved hospital database over a period of 4 years from 1st January 2018 to 31st December 2022.

### Population selection

All patients with a diagnosis acute bronchiolitis hospitalised in any of the three hospitals during the study period were eligible for the study. We included only patient aged 1–24 months. Patients with a diagnosis of asthma or who had 2 previous episodes of acute bronchiolitis were excluded. Records with missing data on demographic characteristics like age and sex as well as outcomes were also excluded.

### Outcome of interest

The main outcome of interest was prolonged hospital length of stay (LOS). Prolonged hospital stay was defined as at least one hospital stay of 5 or more days in a paediatric unit for acute bronchiolitis [18]. The secondary outcome of interest was death during hospitalisation.

### Predictor of interest

The main predictors of interest were age, and initial oxygen saturation [18].

### Sample size calculation

Rodriguez et al. [18] reported that mean initial oxygen saturation (%) was 86.4 ± 5.4SD in patients with prolonged hospital LOS while this was 88.4 ± 5.0SD in patients without prolonged hospital LOS. Based on these results, for a desired confidence interval of 95%, a power of 80%, the ratio of exposed samples to those not exposed of 1, a significance threshold of 5%, the minimum sample size is 107 patients in each exposure group, giving a total of 214 patients with acute bronchiolitis [19].

### Data collection tools and procedures

Medical records of patients hospitalized for bronchiolitis were reviewed for the following demographic, clinical, and microbiologic information: month of admission, age in months, gender (male, female), presence of undernutrition and other comorbidities like trisomy 21, congenital heart disease (yes/no), initial peripheral oxygen saturation (%), and previous episode of bronchiolitis (yes/no). Data was collected using a pretested data collection form and entered in excel sheet for storage.

### The diagnosis of acute bronchiolitis

Acute bronchiolitis was diagnosed based on history and physical exams alone as per recommendations [20]. Clinical diagnosis of acute bronchiolitis was retained in any infant presenting with any first episode of wheezing preceded by or associated with two-to-three-day viral prodrome of fever, cough, rhinorrhoea, and a variable degree of respiratory distress. Any second episode of wheezing with personal or familial history of atopy received a trial of salbutamol. If there was a clinically relevant response this was classified as asthma and if no response, it was classified as bronchiolitis [21]. Most if not all paediatric units in Cameroon, RSV rapid tests are not available and therefore no infant hospitalized for acute lower respiratory infections is tested for RSV.

The hospitalisation criteria for patients with acute bronchiolitis are: significant deterioration in general condition, apnea, cyanosis, respiratory rate > 60, age ≤ 6 weeks, history of prematurity ˂ 34 weeks of amenorrhea, corrected age of ˂3 months, underlying heart disease, transcutaneous arterial oxygen saturation below 94%, digestive disorders compromising hydration, psychosocial difficulties, ventilatory disorder on chest x-ray performed in the face of clinical arguments [15].

### Management guidelines of acute bronchiolitis

In September 2000, a consensus conference (CC) organised by the HAS (Haute Autorité de Santé) in France recommended diagnostic and management guidelines for acute bronchiolitis [11]. Though these management guidelines have been updated in November 2019, both guidelines recommend comprehensive symptomatic therapy, with emphasis on nasopharyngeal wash (NPW) and prohibit the use of antibiotics (unless in cases when secondary bacterial infection is confirmed), corticosteroid therapy, or bronchodilators [13]. In Cameroon, no local management guidelines have been established and as such many consultants practicing in predominantly French-speaking cities like Douala, use guidelines from France. Our reference mainly relates to the guidelines from 2000 because our study covered hospitalisations in 2018–2019, which were prior to the release of these updated guidelines.

#### Statistical analysis

Data was entered and analysed using the SPSS version 28 software. Quantitative variables were presented as mean and standard deviations, while qualitative variables were presented as frequencies (counts) and percentages. Mean values were compared between patients with and without the outcomes of interest using the independent Student t-test. Multivariable linear regression analysis was then performed using variables with *p* ≤ 0.250 [22] on univariate analysis to identify independent factors associated with the outcomes of interest. Statistical significance was set at *p* ≤ 0.05 and the strength of the association was expressed as odds ratio with the 95% Confidence Interval (CI). Kaplan-Meier curves was displayed to depict survival time based on the most significant predictor of mortality event with subsequent log-rank test analysis.

#### Ethical considerations

Ethical clearance was obtained from the Institutional Ethics Committee for Research on Human Health of the University of Douala No 3527 CEI-UDo/03/2023/T. Our study was carried out in strict compliance with the fundamental principles of medical research.

### Operational definitions

#### Fever

Temperature greater than or equal to 38 degrees.

#### Tachycardia

Heart rate greater than 180 beats per minute for patients less than 12 months [23] and above 140 beats per minute for those aged 13–24 months [24].

#### Abnormal respiratory rate

This is a broad term that refers to any respiratory rate that is not within normal limits i.e., 30–60 breaths per minute. Tachypnea referred to respiratory rate above 60 breaths per minute [23].

#### Hypoxia

Peripheral oxygen saturation below 94% [11].

#### Leukocytosis

White blood cell counts above 20 × 10^6^ cells/uL [25].

#### Thrombocytosis

Platelet count above 450 × 10^6^ cells/uL [26].

#### Severe anaemia

Haemoglobin level below 7 g/dL.

#### Undernutrition

Because of the retrospective nature of the study, only weight and age were correctly reported in files. We therefore considered undernutrition as any patient with Weight-for-Age Z scores below − 2.

#### Non-adherence to guidelines

Based on the CC guidelines, non-adherence to guidelines was considered if a patient received any of the following treatments: bronchodilators, corticosteroids, chest physiotherapy, nebulization with saline and antibiotics without evidence of alveolar infiltrates or leukocytosis [15, 27].

#### Oxygen supplementation targets

Although most updated guidelines recommend < 90% as target for oxygen supplementation [6], the pediatric department at Laquintinie has adopted the level of < 94% as a safeguard measure. This is because some authors have reported that pulse oximetry overestimates oxygen saturation amongst blacks patients [28, 29].

#### Seasons

Dry season refers to the period running from November-February and July-August each year while the rainy season runs from March-June and September-October each year [30].

## Results

### Patient recruitment

In total, 7314, 6434 and 3653 patients were hospitalised at HLD, HGD and HGOPED respectively during the study period, of which 215 cases were diagnosed of acute bronchiolitis (HLD = 85, HGD = 59 and HGOPED = 71). The overall prevalence of acute bronchiolitis in these three major paediatric hospitals was 1.24% (*n* = 215/17,401) representing 1.2%, 0.92% and 1.94% for HLD, HGD and HGOPED respectively.

### Sociodemographic characteristics of patients and seasonal variations

**Fig. 1:**
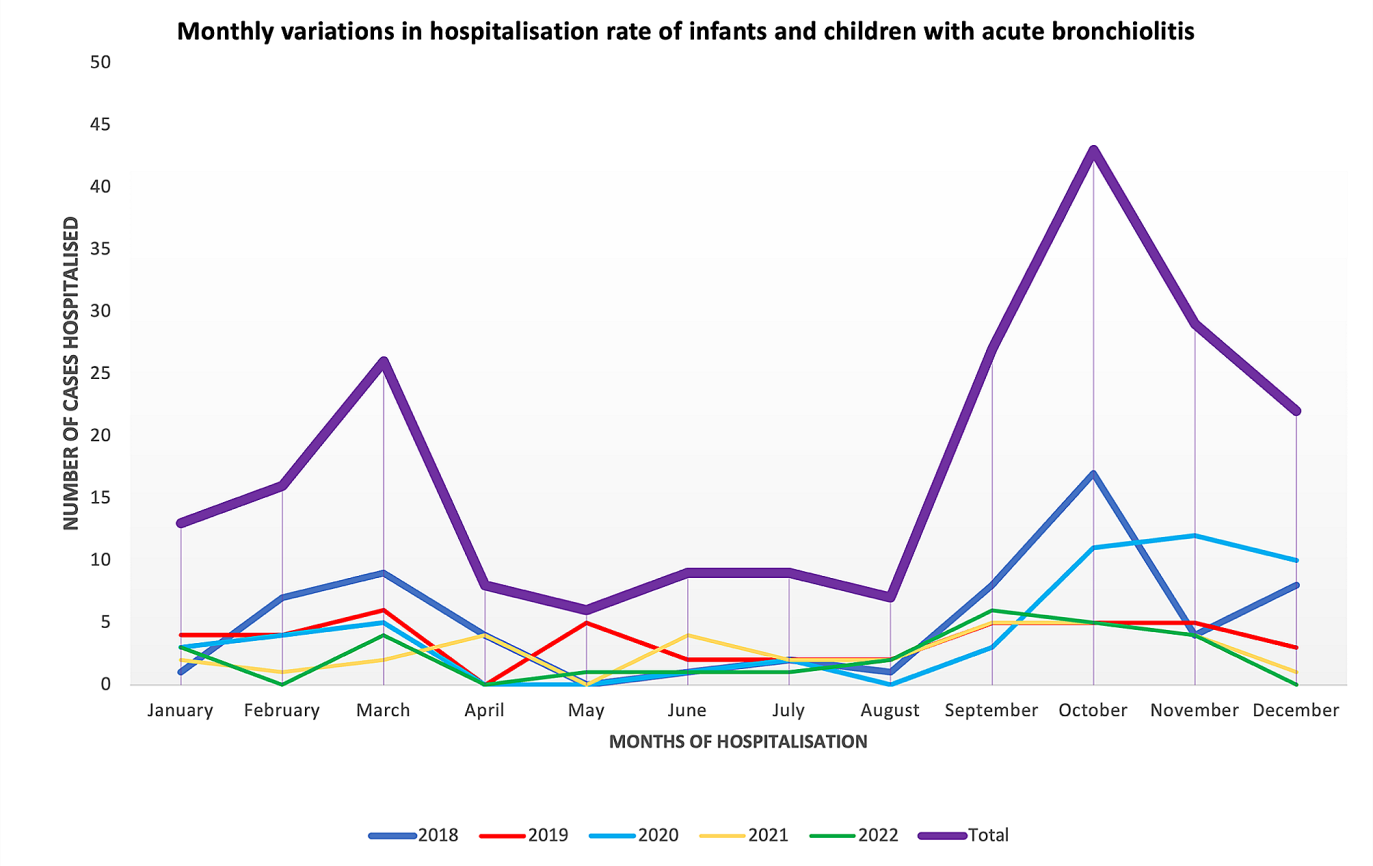
Monthly variations in acute bronchiolitis cases over the study period

Mean age of patients was 6.94 ± 5.71 months. Most represented age group was 3–11 months (*n* = 125, 58.14%) followed by less than 3 months (*n* = 49, 22.79%) and the 12–24 months (*n* = 41, 19.07%). Males constituted 58.14% (*n* = 125) of the sample while females made up 41.86% (*n* = 90) with a M/F sex ratio of 1.39. Most cases were hospitalised during the rainy season (*n* = 119, 55.35%) with peak hospitalisation months being March and October. These trends have been similar over the years as seen in Fig. 1.

### Prehospital management

Prior to admission, patients received antipyretics in 30.23% (*n* = 65), antibiotics in 26.05% (*n* = 56), cough suppressants in 3.26% (*n* = 7), and mucolytics in 3.26% (*n* = 7) of cases. Symptoms prior to admission included cough (*n* = 146, 67.91%), difficulty breathing (*n* = 93, 43.26%), refusal to feed (*n* = 74, 34.42%), rhinorrhoea (*n* = 74, 34.42%), vomiting (*n* = 23, 10.7%) and diarrhoea (*n* = 16, 7.44%) as shown in Table I.

**Table 1:** Comparison of selected variables between patients with or without prolonged hospitalisation

Variable	Prolonged hospitalizations (*n* = 101)	Without prolonged hospitalizations (*n* = 114)	Total	*p* value
Age in months, mean ± SD	7.04 ± 5.94	6.87 ± 5.51	6.94 ± 5.71	0.834
Gender, male/female	53/48	72/42	125/90	**0.113**
Previous episode of bronchiolitis	5 (4.95)	13 (11.4)	18 (8.37)	**0.088**
Undernutrition	18 (17.82)	21 (18.42)	39 (18.14)	0.909
Presenting complaint				
Refusal to feed	37 (36.63)	37 (32.46)	74 (34.42)	0.520
Cough	76 (75.25)	70 (61.40)	146 (67.91)	**0.030**
Dyspnea	58 (57.43)	64 (56.14)	93 (43.26)	0.849
Diarrhoea	13 (12.87)	3 (2.63)	16 (7.44)	**0.004**
Rhinorrhea	33 (32.67)	41 (35.96)	74 (34.42)	0.612
Fever	52 (51.49)	61 (53.51)	113 (52.56)	0.797
Hypoxia	67 (66.34)	60 (52.63)	127 (59.07)	**0.041**
Tachycardia	56 (55.45)	81 (71.05)	137 (63.72)	**0.018**
Abnormal respiratory rate	31 (30.69)	17 (14.91)	48 (22.33)	**0.069**
Alveolar opacities on CXR (*n* = 37/77)	7/15 (46.67)	11/22 (50.00)	18/37 (48.65)	0.842
Adherence to guidelines	1 (0.99)	9 (7.89)	10 (4.65)	**0.016**
Leukocytosis > 20x x10^6^	5 (6.33)	9 (10.71)	14 (8.59)	0.607
Severe anemia	3 (3.85)	1 (1.2)	4 (2.48)	0.282
Thrombocytosis	25 (29.76)	23 (29.11)	48 (29.45)	0.991
Oxygen administered	80 (79.21)	66 (57.89)	146 (67.91)	**0.001**

Bold values represent variables with statistical significance for inclusion in logistic regression analysis

### Clinical characteristics

On admission, 3.26% (*n* = 7) of our patients had cyanosis, use of accessory muscles of respiration in 16.28% (*n* = 35), nasal flaring in 66.98% (*n* = 144), grunting in 33.49% (*n* = 72), thoraco-abdominal asynchrony in 44.19% (*n* = 95), intercostal recession in 60.74% (*n* = 130). Coarse and fine crackles were present in 48.84% (*n* = 105), rhonchi in 37.67% (*n* = 81). Silent chest was reported in one patient.

Mean initial temperature was 38.06 ± 0.94 SD ºC, mean initial peripheral oxygen saturation was 90.28 ± 9.99 SD %, mean initial heart rate was 145.9 ± 26.56 SD beats per minute and mean initial respiratory rate was 49.55 ± 15.48 SD breaths per minute. A total of 52.56% (*n* = 113) had fever, 59.97% (*n* = 127) had hypoxia, 63.72% (*n* = 137) had tachycardia, and 23.3% (*n* = 48) had abnormal respiratory rate as shown in Table I. Up to 34.42% of patients had comorbidities (*n* = 74) which were polymalformative syndromes (*n* = 2, 2.7%), congenital heart diseases (*n* = 12, 16.22%), trisomy 21 (*n* = 3, 4.95%), acute undernutrition (*n* = 39, 18.14%) and one case each of prematurity, laryngomalacia and HIV (*n* = 1, 1.35%).

### In-hospital management

Corticosteroids were received in 75.81% (*n* = 163), antibiotics in 74.88% (*n* = 161). and salbutamol nebulization in 78.6% (*n* = 169). Nasal lavage was performed in 92.09% (*n* = 198), saline nebulization in 44.19% (*n* = 95), respiratory physiotherapy in 13.46% (*n* = 29), and nasogastric tube feeding in 35.35% (*n* = 76). About 5/16 (31.25%) children who had diarrhea in history of presenting complaint received IV fluid rehydration.

### Laboratory and radiology results

Mean initial white blood cell count was 12.47 ± 6.74 SD x10^6^ cells/uL, mean initial hemoglobin level was 10.4 ± 1.79 SD g/dL and mean initial platelet count was 371.76 ± 171.44 SD x10^3^ cells/uL. Leukocytosis was present in 8.59% of patients, severe anemia in 2.48% and thrombocytosis in 29.45%. Chest X-ray was requested in 35.81% (*n* = 77/215) of patients and was abnormal in 48.05% (*n* = 37/77) of them. Alveolar opacities were present in 48.64% (*n* = 18/37) cases who performed a Chest X-ray as shown in Table I.

### Factors associated with prolonged length of stay (LOS) and mortality

Prolonged LOS was noted in 46.97% (*n* = 101) and mortality in 10.7% (*n* = 23) of patients. Mortality rate per age group was 16.33% (*n* = 8/49), 10.4% (*n* = 13/125) and 4.88% (*n* = 2/41) in patients aged less than 3 months, 3–11 months, and 12–24 months respectively. Prematurity, laryngomalacia, HIV and congenital heart diseases were insufficiently represented in outcome categories and therefor no sub analysis was conducted.

On univariate analysis, factors associated with prolonged hospital stay were female gender, previous episode of bronchiolitis, cough, diarrhoea, hypoxia, tachycardia, non-adherence to guidelines and oxygen administration as shown in Table I. On multivariable analysis, factors independently associated with prolonged hospital duration were oxygen administration [b = 0.36, OR = 2.35 (95% CI:1.16–4.74), *p* = 0.017], abnormal respiratory rate [b = 0.38, OR = 2.13 (1.00-4.55), *p* = 0.050] and patients presenting with cough [b = 0.33, OR = 2.35 (95% CI: 1.22–4.51), *p* = 0.011], and diarrhoea [b = 0.71, OR = 6.44 (95% CI: 1.6-25.86), *p* = 0.009] on admission as shown in Table II.

**Fig. 2:**
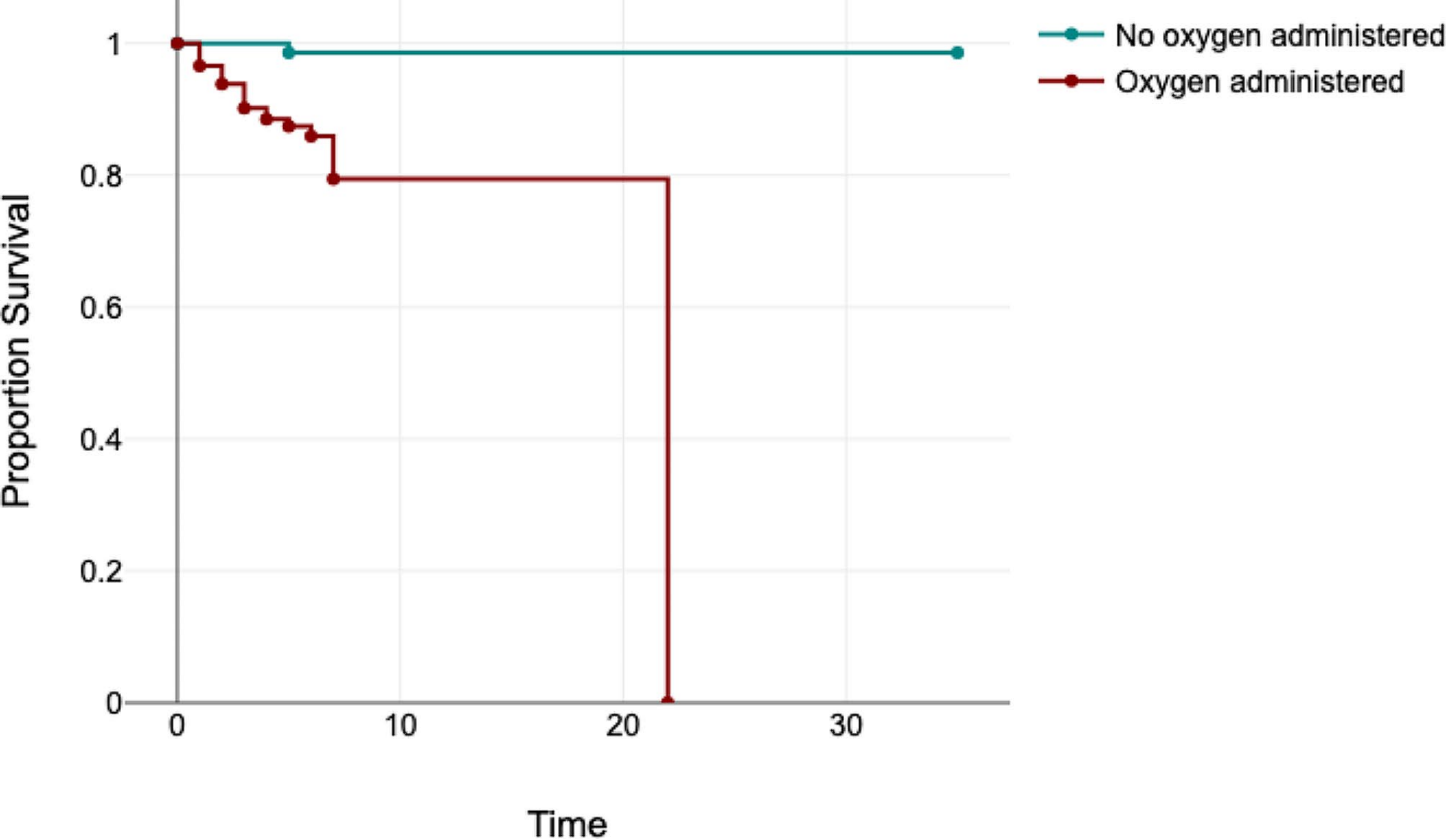
Kaplan-Meier survival curve displaying mortality event rates over hospitalisation days (time) based on oxygen administration status

Factors associated with mortality on univariate analysis were age of the patient in months, cough as presenting complaint, fever, female gender, tachycardia, and oxygen administration as shown in Table III. On multivariable analysis, factors independently associated with mortality were age of the patient [b= -0.07, OR = 0.84 (95% CI: 0.74–0.97), *p* = 0.014] and oxygen administration [b = 1.08, OR = 9.64 (95% CI:1.16–79.85), *p* = 0.036] as shown in Table IV. A Kaplan-Meier survival curve was used to depict survival rates based on oxygen administration in Fig. 2. The log-rank test showed that there is a difference between oxygen administration status in terms of the distribution of time until the mortality event occurs, *p* = 0.009.

**Table 2:** Predictors of prolonged hospital duration on logistic regression analysis

Variable	OR (95% CI)	*p* value	b	aOR (95% CI)	*p* value
Female gender	1.55 (0.9–2.68)	**0.114**	0.31	1.49 (0.81–2.74)	0.200
Tachycardia	0.51 (0.29–0.89)	**0.018**	0.32	0.59 (0.31–1.120	0.105
Hypoxia	1.77 (1.02–3.08)	**0.042**	0.33	1.72 (0.9–3.28)	0.103
Cough as presenting complaint	1.91 (1.06–3.44)	**0.024**	0.33	2.35 (1.22–4.51)	**0.011**
Oxygen administered	2.77 (1.51–5.09)	**0.001**	0.36	2.35 (1.16–4.74)	**0.017**
Abnormal respiratory rate	1.85 (0.95–3.57)	**0.071**	0.38	2.13 (1.00-4.55	**0.050**
Previous episode of bronchiolitis	0.4 (0.14–1.18)	**0.097**	0.62	0.48 (0.14–1.61)	0.235
Diarrhoea as presenting complaint	5.47 (1.51–19.78)	**0.010**	0.71	6.44 (1.6-25.86)	**0.009**
Non-adherence to guidelines	8.57 (1.07–68.89)	**0.043**	1.1	4.87 (0.56–42.17)	0.151
Corticosteroids used	1.17 (0.41–3.31)	0.772	-	-	-
Antibiotics used^*^	2.41 (0.69–8.46)	**0.169**	-	-	-
Salbutamol nebulization used^*^	6.73 (0.88–51.36)	**0.066**	-	-	-

^*^Not included in the model because these variables are composite measures of the variable non-adherence to guideline. OR = Odd’s ratio. CI = Confidence interval. aOR = Adjusted Odd’s ratio

**Table 3:** Comparison of selected variables between patients alive and dead

Variable	Patients alive (*n* = 192)	Patient who died (*n* = 23)	Total (*n* = 215)	*p* value
Age in months, mean ± SD	7.42 ± 5.86	4.42 ± 3.37	6.94 ± 5.71	0.025
Gender, male/female	109/83	16/7	125/90	**0.240**
Previous episode of bronchiolitis	16 (8.33)	2 (8.7)	18 (8.37)	0.953
Undernutrition	35 (18.23)	4 (17.39)	39 (18.14)	0.922
Presenting complaint				
Refusal to feed	68 (35.42)	6 (26.09)	74 (34.42)	0.373
Cough	134 (69.79)	12 (52.17)	146 (67.91)	**0.087**
Dyspnea	82 (42.71)	11 (47.83)	93 (43.26)	0.640
Diarrhoea	16 (8.33)	-	16 (7.44)	**0.150**
Rhinorrhea	64 (33.3)	10 (43.48)	74 (34.42)	0.333
Fever	98 (51.04)	15 (65.22)	113 (52.56)	**0.198**
Hypoxia	114 (59.38)	13 (56.52)	127 (59.07)	0.793
Tachycardia	119 (61.98)	18 (78.26)	137 (63.72)	**0.125**
Abnormal respiratory rate	41 (21.35)	7 (30.43)	48 (23.33)	0.323
Alveolar opacities on CXR (*n* = 37/77)	17/35 (48.57)	1/2 (50.00)	18/37 (48.65)	0.258
Adherence to guideline	10 (5.2)	-	10 (4.6)	0.262
Corticosteroids used	145 (75.52)	18 (78.26)	163 (75.81)	0.772
Antibiotics used	141 (73.44)	20 (86.96)	161 (74.88)	**0.158**
Salbutamol nebulization used	147 (76.56)	22 (95.65)	169 (78.6)	**0.035**
Leukocytosis > 20x x10^6^	12 (8.28)	2 (11.11)	14 (8.59)	0.686
Severe anemia	4 (2.8)	-	4 (2.48)	0.472
Thrombocytosis	44 (30.34)	4 (22.22)	48 (29.45)	0.748
Oxygen administered	124 (64.58)	22 (95.65)	146 (67.91)	**0.003**

CXR = Chest X-ray. SD = Standard Deviation. Bold values represent variables with statistical significance

**Table 4:** Predictors of mortality on logistic regression analysis

Variable	OR (95% CI)	*p* value	b	aOR (95% CI)	*p* value
Age of the patient in months	0.88 (0.78–0.99)	**0.031**	0.07	0.84 (0.74–0.97)	**0.014**
Cough as presenting complaint	0.47 (0.2-1,13)	**0.093**	0.50	0.69 (0.26–1.84)	0.454
Fever	1.8 (0.73–4.44)	**0.203**	0.54	2.65 (0.93–7.57)	0.069
Female gender	0.57 (0.23–1.46)	**0.244**	0.52	0.56 (0.20–1.55)	0.266
Tachycardia	2.21 (0.79–6.2)	**0.133**	0.57	2.94 (0.95–9.06)	0.061
Oxygen administered	12.06(1.59–91.47)	**0.016**	1.08	9.64 (1.16–79.85)	**0.036**
Antibiotics used	4.96 (2.38–10.3)	**< 0.001**	0.73	1.19 (0.29–4.96)	0.812
Salbutamol nebulization used	1.9 (0.96–3.74)	**0.064**	1.09	7.87 (0.93–66.33)	0.058

## Discussion

The predominance of male gender in our study has been reported before in Brazil, Senegal and Cameroon [1, 18, 31]. Though it is commonly recognised that females have smaller airways [32], regardless of age, females have been reported to have stronger antibody responses, baseline immunoglobulin levels, and B cell counts than males [33, 34]. Moreover, the Single Nucleotide Polymorphisms or SNP (rs2069885) in the interleukin (IL)-9 gene which has been associated with severe RSV infection requiring hospitalisation is associated with an increased susceptibility in boys [35]. All these factors may explain why males are more affected by respiratory infections than females [36, 37].

Bronchiolitis is caused by inflammation of the lining of the epithelial cells of the small airways of the lungs, which results mucus production, inflammation, and cellular necrosis [38]. This inflammation can restrict the airway and eventually cause cough, wheezing. and **a**bnormal respiratory rate. These symptoms are less common in mild forms of the disease and therefore are surrogate measures of severity [4]. This may explain why cough and abnormal respiratory rate were associated with prolonged hospital stay in our study.

The administration of oxygen was the single most important factor associated with both prolonged LOS and mortality. It is well recognised that tissue hypoxia can lead to cell death. However, hyperoxaemia can also be dangerous for lung tissues [39]. Arterial PCO2 plays an important role in respiratory drive and therefore, high oxygen concentrations can compromise respiratory centre function and aggravate hypercapnia [40]. Oxygen administration must therefore be carefully codified, even in resource limited settings like ours. According to the Consensus Conference (CC) guidelines, all patients with peripheral oxygen saturation below 94% should be hospitalised and given oxygen [11]. Nonetheless, because of the potential dangers of excessive oxygen administration, increased cost of care and the potential increased risk in the transmission of nosocomial infections using oxygen delivery devices, some authors have proposed lower targets for oxygen saturation [41–43]. Cunningham et al. [41], in a randomised controlled trial reported that the time taken for symptoms to resolve was the same whether in patients with acute bronchiolitis whether managed with a target oxygen saturation of 90% or 94%. Nonetheless, we believe that the main reason why oxygen administration was a significant factor associated with mortality and prolonged LOS in our study was because it is a marker of disease severity [44]. More studies are necessary in our setting to validate adequate targets of oxygen saturation in the management of acute bronchiolitis in infants and young children.

Diarrhea was present in 7.44% of our patients. It causes significant fluid losses, which in addition to fluid losses from increased respiratory efforts can predispose infants and children to clinically significant dehydration. Due to the retrospective nature of our study, dehydration was not evaluated. Nonetheless, we hypothesise that diarrhea was associated with prolonged LOS because it led to dehydration in these patients. Dehydration has been reported previously to increased LOS in patients with acute bronchiolitis [45],

In our study, an increase in age of the patient (in months) was associated with a decrease in the probability of mortality. Though the highest age group affected was 3–11 months, the highest mortality rate was reported in patients aged less than 3 months (16.33%). This finding has been similarly reported in other studies [46, 47] and is attributed to the rapid disease progression and immune fragility of younger infants [48, 49]. Because of these findings, some authors have suggested that vaccination strategies be focused on infants in their early life [50]. Nonetheless, other authors argue that this finding may represent survival bias as common risk factors for death like prematurity and neonatal infections might create a survival disadvantage at this age [51]. This is especially true in our study as we did not explore the contribution of prematurity, laryngomalacia, HIV and congenital heart diseases in these overall deaths.

In our study, 95.35% of the patients received either corticosteroids, antibiotics, or salbutamol nebulization, translating into poor adherence to management guidelines in only 4.65% of cases. Despite the fact that several authors [52, 53] and expert societies [14, 20] have reported that corticosteroids have no added benefits in the management of acute bronchiolitis, it was administered in more than 70% of patients in this study. According to an Italian study, four out of ten paediatricians are aware of recommended diagnostic criteria for acute bronchiolitis, however, 39.64% still prescribe bronchodilators, while 64.52% prescribe systemic corticosteroids [54]. The apparent severity, the associated anxiety from treating such severely ill children without medications, and personal reassurance or parental pressure are the reasons stated for this wrong practice [55]. Continuous Medical Education (CME) sessions need to be organised to harmonize practices in the management of acute bronchiolitis in Cameroon.

The study period spanning from 2018 to 2022 includes the period of the COVID-19 pandemic in Cameroon (1st case reported in March 2020). This could have contributed significantly in underestimating the real prevalence of acute bronchiolitis in our setting, as up to 27–47% drop in pediatric hospitalisation frequency in Cameroon has been reported [56]. This study was limited by its retrospective nature that prevented us from exploring all potential explanatory data like hydration status and prematurity. Moreover, mortality from RSV bronchiolitis has been reported to be different from mortality from other viral-induced bronchiolitis [18] and therefore, lack of viral testing in our study was a significant limitation. Moreover, although sometimes reported in all patients aged < 24 months [57], bronchiolitis typically affects infants aged 1-12months. Our sample also included patients aged 12–24 months and therefore our results may represent RSV induced Lower Respiratory Tract Infections (LRTI)) rather than bronchiolitis. A nationwide prospective study should be conducted to address the methodological flaws of this current study and explore nationwide trends in prevalence, and factors associated with hospitalisation, length of hospital stay and mortality.

## Conclusion

Acute bronchiolitis represented 1.24% of pediatric hospitalisations, with peak admissions during the rainy season, particularly in March and October and this was consistent over the years. The most affected age group was 3–11 months, with males being more commonly affected. Management guidelines were poorly respected. Prolonged length of stay was reported in half of the patients and associated with oxygen administration, abnormal respiratory rate, cough, and diarrhea on admission. Mortality was high, with one out of ten patients dying, especially younger patients and patients receiving oxygen. Adequate implementation of management strategies may reduce hospital stays and improve outcomes for patients with acute bronchiolitis.

## Data Availability

The data that support the findings of this study are available on request from the corresponding author, CEE.

## Abbreviations

LOS: Length of stay
SD: Standard Deviation
HIV: Human Immunodeficiency Virus

## References

[1] Seck N, Basse I, Keïta Y, Boiro D, Thiam L, Ndongo AA (2018). La Bronchiolite aiguë Du nourrisson en milieu tropical. J Pédiatrie Puériculture.

[2] Freymuth F, Vabret A, Dina J, Cuvillon-Nimal D, Lubin C, Vaudecrane A (2010). Les virus des bronchiolites aiguës. Arch Pédiatrie.

[3] Kenmoe S, Kengne-Nde C, Ebogo-Belobo JT, Mbaga DS, Modiyinji AF, Njouom R (2020). Systematic review and meta-analysis of the prevalence of common respiratory viruses in children < 2 years with bronchiolitis in the pre-COVID-19 pandemic era. PLoS ONE.

[4] Amat F, Henquell C, Verdan M, Roszyk L, Mulliez A, Labbé A (2014). Predicting the severity of acute bronchiolitis in infants: should we use a clinical score or a biomarker?. J Med Virol.

[5] Heppe Montero M, Gil-Prieto R, Walter S, Aleixandre Blanquer F, De Gil Á. Burden of severe bronchiolitis in children up to 2 years of age in Spain from 2012 to 2017. Hum Vaccines Immunother. 18:1883379. 10.1080/21645515.2021.1883379 PMC8920124 33653212

[6] Manti S, Staiano A, Orfeo L, Midulla F, Marseglia GL, Ghizzi C (2023). UPDATE – 2022 Italian guidelines on the management of bronchiolitis in infants. Ital J Pediatr.

[7] Kramer R, Duclos A, Lina B, Casalegno JS, VRS study group in Lyon (2018). Cost and burden of RSV related hospitalisation from 2012 to 2017 in the first year of life in Lyon. France Vaccine.

[8] RP_190_RG_Mercier.pdf [. Internet]. [cité 2022 déc 13];Available from: http://www.realites-pediatriques.com/wp-content/uploads/sites/3/2016/04/RP_190_RG_Mercier.pdf.

[9] Bogne JB, Chiabi A, Tchatat DY, Nguefack S, Mah E, Tchokoteu PF et al. Bronchiolite Aiguë du Nourrisson de Moins de 24 mois à Yaoundé (À Propos de 296 Cas). Health Sci. Dis. [Internet]. 2013 [cité 2022 nov 29];14. Available from: http://www.hsd-fmsb.org/index.php/hsd/article/view/220.

[10] Doumbia A, Togo P, Coulibaly O, Dembélé A, Sacko K, Maiga B et al. La bronchiolite aiguë du nourrisson: à propos de 112 cas hospitalisés au département pédiatrie du CHU Gabriel Touré. Rev. Malienne Infect. Microbiol. [Internet] 2018 [cité 2023 janv 6];Available from: https://revues.ml/index.php/remim/article/view/985.

[11] Stagnara J, Balagny E, Cossalter B, Dommerges JP, Dournel C, Drahi E (2001). Prise en charge de la bronchiolite du nourrisson texte court. Arch Pédiatrie.

[12] Subcommittee on Diagnosis and Management of Bronchiolitis (2006). Diagnosis Manage Bronchiolitis Pediatr.

[13] Haute Autorité de Santé, Conseil National Professionel (CNP) de Pediatrie. Les recommandations de bonne pratique (RBP): Prise en charge du premier épisode de bronchiolite aiguë chez le nourrisson de moins de 12 mois [Internet]. 2019;Available from: https://www.has-sante.fr/upload/docs/application/pdf/2019-11/hascnpp_bronchiolite_texte_recommandations_2019.pdf.

[14] David M, Luc-Vanuxem C, Loundou A, Bosdure E, Auquier P, Dubus JC (2010). Application de la Conférence de consensus sur la bronchiolite aiguë du nourrisson en médecine générale: évolution entre 2003 et 2008. Arch Pédiatrie.

[15] Branchereau E, Branger B, Launay E, Verstraete M, Vrignaud B, Levieux K (2013). État des lieux des pratiques médicales en médecine générale en matière de bronchiolite et déterminants de prises en charge thérapeutiques discordantes par rapport aux recommandations de l’HAS. Arch Pédiatrie.

[16] Demont C, Petrica N, Bardoulat I, Duret S, Watier L, Chosidow A (2021). Economic and disease burden of RSV-associated hospitalizations in young children in France, from 2010 through 2018. BMC Infect Dis.

[17] Maki K, Azizi H, Hans P, Doan Q (2020). Adherence to national paediatric bronchiolitis management guidelines and impact on emergency department resource utilization. Paediatr Child Health.

[18] Rodríguez-Martínez CE, Sossa-Briceño MP, Nino G (2018). Predictors of prolonged length of hospital stay for infants with bronchiolitis. J Investig Med off Publ Am Fed Clin Res.

[19] Dean AG, Sullivan KM, Soe MM, OpenEpi. Open Source Epidemiologic Statistics for Public Health, Version 3.01 [Internet]. 2013 [cité 2022 avr 12];Available from: www.OpenEpi.com.

[20] Friedman JN, Rieder MJ, Walton JM, Bronchiolitis (2014). Recommendations for diagnosis, monitoring and management of children one to 24 months of age. Paediatr Child Health.

[21] Benhamida M, Bihouee T, Verstraete M, Guen CGL, Launay E (2017). Retrospective audit of guidelines for investigation and treatment of bronchiolitis: a French perspective. BMJ Paediatr Open.

[22] Grant SW, Hickey GL, Head SJ (2019). Statistical primer: multivariable regression considerations and pitfalls†. Eur J Cardiothorac Surg.

[23] Prise en charge du 1er épisode de. bronchiolite aiguë chez le nourrisson de moins de 12 mois [Internet]. Haute Aut. Santé [cité 2023 oct 9];Available from: https://www.has-sante.fr/jcms/p_3118113/fr/prise-en-charge-du-1er-episode-de-bronchiolite-aigue-chez-le-nourrisson-de-moins-de-12-mois.

[24] Fleming S, Thompson M, Stevens R, Heneghan C, Plüddemann A, Maconochie I (2011). Normal ranges of heart rate and respiratory rate in children from birth to 18 years: a systematic review of observational studies. Lancet.

[25] Girodias JB, Bailey B (2003). Approach to the febrile child: a challenge bridging the gap between the literature and clinical practice. Paediatr Child Health.

[26] Rokkam VR, Killeen RB, Kotagiri R. Secondary Thrombocytosis [Internet]. In: StatPearls. Treasure Island (FL): StatPearls Publishing; 2023 [cité 2023 oct 9]. Available from: http://www.ncbi.nlm.nih.gov/books/NBK560810/. 32809645

[27] Roqué-Figuls M, Giné-Garriga M, Rugeles CG, Perrotta C, Vilaró J. Chest physiotherapy for acute bronchiolitis in paediatric patients between 0 and 24 months old. Cochrane Database Syst. Rev. [Internet]. 2023 [cité 2023 nov 14];Available from: https://www.cochranelibrary.com/cdsr/doi/10.1002/14651858.CD004873.pub6/full. 10.1002/14651858.CD004873.pub6 PMC10070603 37010196

[28] Fawzy A, Wu TD, Wang K, Robinson ML, Farha J, Bradke A (2022). Racial and ethnic discrepancy in pulse oximetry and delayed identification of treatment eligibility among patients with COVID-19. JAMA Intern Med.

[29] Andrist E, Nuppnau M, Barbaro RP, Valley TS, Sjoding MW (2022). Association of Race with Pulse Oximetry Accuracy in Hospitalized Children. JAMA Netw Open.

[30] Lontchi-Yimagou E, Tsalefac M, Tapinmene LMT, Noubiap JJN, Balti EV, Nguewa JL (2016). Seasonality in diabetes in Yaounde, Cameroon: a relation with precipitation and temperature. BMC Public Health.

[31] Kalla GCM, Momo YCB, Noubi NK, Mandeng MNE, Monebenimp F (2020). Déterminants De La durée prolongée d’hospitalisation chez les nourrissons âges de 1 à 24 mois admis pour bronchiolite aiguë dans trois hôpitaux de Yaoundé. Rev Médecine Pharm.

[32] Molgat-Seon Y, Peters CM, Sheel AW (2018). Sex-differences in the human respiratory system and their impact on resting pulmonary function and the integrative response to exercise. Curr Opin Physiol.

[33] Klein SL, Flanagan KL (2016). Sex differences in immune responses. Nat Rev Immunol.

[34] Ursin RL, Klein SL (2021). Sex differences in respiratory viral pathogenesis and treatments. Annu Rev Virol.

[35] Schuurhof A, Bont L, Siezen CLE, Hodemaekers H, van Houwelingen HC, Kimman TG (2010). Interleukin-9 polymorphism in infants with respiratory syncytial virus infection: an opposite effect in boys and girls. Pediatr Pulmonol.

[36] Dias SP, Brouwer MC, van de Beek D (2022). Sex and gender differences in bacterial infections. Infect Immun.

[37] Jacobsen H, Klein SL (2021). Sex differences in immunity to viral infections. Front Immunol.

[38] Erickson EN, Bhakta RT, Mendez MD. Pediatric Bronchiolitis [Internet]. In: StatPearls. Treasure Island (FL): StatPearls Publishing; 2023 [cité 2023 oct 11]. Available from: http://www.ncbi.nlm.nih.gov/books/NBK519506/. 30137791

[39] Cooper JS, Phuyal P, Shah N. Oxygen Toxicity [Internet]. In: StatPearls. Treasure Island (FL): StatPearls Publishing; 2023 [cité 2023 oct 11]. Available from: http://www.ncbi.nlm.nih.gov/books/NBK430743/.

[40] Brinkman JE, Toro F, Sharma S, Physiology. Respiratory Drive [Internet]. In: StatPearls. Treasure Island (FL): StatPearls Publishing; 2023 [cité 2023 oct 11]. Available from: http://www.ncbi.nlm.nih.gov/books/NBK482414/. 29494021

[41] Cunningham S, Rodriguez A, Adams T, Boyd KA, Butcher I, Enderby B (2015). Oxygen saturation targets in infants with bronchiolitis (BIDS): a double-blind, randomised, equivalence trial. Lancet Lond Engl.

[42] Napolitano N, Berlinski A, Walsh BK, Ginier E, Strickland SL (2021). AARC Clinical Practice Guideline: management of Pediatric patients with oxygen in the Acute Care setting. Respir Care.

[43] Ralston SL, Lieberthal AS, Meissner HC, Alverson BK, Baley JE, Gadomski AM (2014). Clinical practice Guideline: the diagnosis, management, and Prevention of Bronchiolitis. Pediatrics.

[44] Rojas-Reyes MX, Granados Rugeles C, Charry‐Anzola LP (2014). Oxygen therapy for lower respiratory tract infections in children between 3 months and 15 years of age. Cochrane Database Syst Rev.

[45] Walsh P, Rothenberg SJ, O’Doherty S, Hoey H, Healy R (2004). A validated clinical model to predict the need for admission and length of stay in children with acute bronchiolitis. Eur J Emerg Med.

[46] Gil-Prieto R, Gonzalez-Escalada A, Marín-García P, Gallardo-Pino C, Gil-de-Miguel A (2015). Respiratory Syncytial Virus Bronchiolitis in Children up to 5 years of age in Spain: epidemiology and comorbidities. Med (Baltim).

[47] Du Y, Yan R, Wu X, Zhang X, Chen C, Jiang D (2023). Global burden and trends of respiratory syncytial virus infection across different age groups from 1990 to 2019: a systematic analysis of the global burden of Disease 2019 study. Int J Infect Dis.

[48] Murphy C, MacLeod WB, Forman LS, Mwananyanda L, Kwenda G, Pieciak RC (2021). Risk factors for respiratory Syncytial virus–Associated Community deaths in Zambian infants. Clin Infect Dis.

[49] Tsafaras GP, Ntontsi P, Xanthou G (2020). Advantages and limitations of the neonatal Immune System. Front Pediatr.

[50] Higgins D, Trujillo C, Keech C (2016). Advances in RSV vaccine research and development - A global agenda. Vaccine.

[51] Gill CJ, Mwananyanda L, MacLeod WB, Kwenda G, Pieciak R, Mupila Z (2022). Infant deaths from respiratory syncytial virus in Lusaka, Zambia from the ZPRIME study: a 3-year, systematic, post-mortem surveillance project. Lancet Glob Health.

[52] Alarcón-Andrade G, Cifuentes L (2018). Should systemic corticosteroids be used for. Bronchiolitis? Medwave.

[53] Fernandes RM, Bialy LM, Vandermeer B, Tjosvold L, Plint AC, Patel H (2013). Glucocorticoids for acute viral bronchiolitis in infants and young children. Cochrane Database Syst Rev.

[54] Manti S, Licari A, Brambilla I, Caffarelli C, Calvani M, Cardinale F (2021). Agreements and controversies of national guidelines for bronchiolitis: results from an Italian survey. Immun Inflamm Dis.

[55] De Brasi D, Pannuti F, Antonelli F, de Seta F, Siani P, de Seta L (2010). Therapeutic approach to bronchiolitis: why pediatricians continue to overprescribe drugs? Ital. J Pediatr.

[56] Chelo D, Mekone Nkwelle I, Nguefack F, Mbassi Awa HD, Enyama D, Nguefack S (2021). Decrease in hospitalizations and increase in deaths during the Covid-19 epidemic in a Pediatric Hospital, Yaounde-Cameroon and Prediction for the coming months. Fetal Pediatr Pathol.

[57] Barbieri E, Cavagnis S, Scamarcia A, Cantarutti L, Bertizzolo L, Bangert M (2023). Assessing the burden of bronchiolitis and lower respiratory tract infections in children ≤ 24 months of age in Italy, 2012–2019. Front Pediatr.

